# Empirical evidence on the ownership and liquidity of real estate tokens

**DOI:** 10.1186/s40854-022-00427-5

**Published:** 2023-01-19

**Authors:** Laurens Swinkels

**Affiliations:** Erasmus Universiteit Rotterdam, Erasmus School of Economics, Rotterdam, Netherlands

**Keywords:** Blockchain, Cryptocurrency, Real estate, Tokenization, G1, G12, G23, G32, K25, O33, R30, R31

## Abstract

To better understand the potential and limitations of the tokenization of real asset markets, empirical studies need to examine this radically new organization of financial markets. In our study, we examine the financial and economic consequences of tokenizing 58 residential rental properties in the US, particularly those in Detroit. Tokenization aims at fragmented ownership. We found that the residential properties examined have 254 owners on average. Investors with a greater than USD 5,000 investment in real estate tokens, diversify their real estate ownership across properties within and across the cities. Property ownership changes about once yearly, with more changes for properties on decentralized exchanges. We report that real estate token prices move according to the house price index; hence, investing in real estate tokens provides economic exposure to residential house prices.

## Introduction

Blockchain technology contains the considerable potential for the tokenization of real assets. However, only a few real assets are available on financial markets in token form.^1^ Hence, literature on the functioning of the tokenization of real assets remains scarce in practice.

To emphasize ownership and liquidity of tokenized financial markets, we utilize a sample of recently tokenized residential properties in the US. From October 2019 to February 2021, RealT—a real estate tokenization firm—has tokenized 58 properties: with 52 tokenized properties in Detroit (MI) and 6 in Chicago (IL), Rochester (NY), Deerfield Beach (FL), Cleveland (OH), Akron (OH), and Dearborn Heights (MI). We selected these 58 properties because these are the only ones that have been tokenized and not because these properties were filtered from a potentially larger set; hence, our sample presents no selection bias. The company’s first tokenized properties are located in Detroit because of the company founders’ familiarity with residential real estate in that area. However, Detroit’s housing market is not easily comparable to that of other states. The global financial crisis of 2007–2008 drove residential house prices down owing to a staggering amount of foreclosures. Mueller and Fontaine (2021) report that the consequences of the crisis can still be felt in the housing market and that mortgage-financed house purchases remain below pre-crisis levels and have almost completely stopped. Seymour and Akers (2022) analyze the causes and consequences of Detroit’s real estate crisis and its effect on the local population.

At the time of digitization, total real estate value was about USD 6.5 million, and about 5% consists of the estimated global market of digitized real estate.^2^ Avoiding a limited sample size is difficult because the practical tokenization of real estate is currently minimal. However, we believe this study examines the only sample of residential real estate properties in existence. Despite this limited sample, we believe a detailed analysis of the ownership distribution of these tokens and its investors’ portfolio choices may provide us with insights into the potential, limitations, and future development of the tokenized real estate market. Savills (2016) estimates the value of total global residential real estate at USD 217 trillion, which exceeds public equity and bond markets combined. However, owing to our small sample size, our findings may not automatically carry over to other tokenization markets nor accurately represent future real estate tokenization.

This research primarily aims to determine whether practical experiences of decentralized finance—particularly that of the tokenization (see Cong and Xiao 2021) of real assets—fulfill its promises. Baum (2021) highlights that fractionalization, customizable ownership diversification, and increased liquidity consists of three important drivers that could drive large-scale adoption of tokenization in the real estate sector. Further asset tokenization may be spurred if the theoretical benefits of tokenization can be realized. We believe our study is the first empirically determine whether major disruptive innovations in the organization of real estate markets can be expected in the future.

We aim to answer the following research questions:

- How concentrated is typical residential property ownership after digitization?
- Do token investors use fractional ownership to diversify their real estate portfolios?
- How liquid are individual residential properties after tokenization?
- Are tokenized asset prices related to the economic fundamentals of the investment?

By attempting to answer these four questions, we contribute to at least three strands of literature. First, we contribute to blockchain literature by adding an empirical dimension to the tokenization of real assets.^3^ Current literature remains abundant with conceptual research on the potential of blockchain technologies to disrupt the organization of financial contracts and markets; see, e.g., Cong and He (2019), Smith et al. (2019), Gupta et al. (2020), Konashevych (2020), Cong et al. (2021), Kou et al. (2021), and Harvey et al. (2021). The initial coin offerings of the real estate properties in our sample are not intended as a source of external financing for projects or firms, as in Howell et al. (2020); however, it is an application of blockchain-based record-keeping as in Yermack (2017). Hence, Taleb’s (2021) criticism of the value of cryptocurrencies is relatively irrelevant here, as tokens represent clear underlying value (the property) and generate cash flows (the rents). The blockchain has been considered fragile, with investors worrying about their wallets being hacked and their tokens being stolen; hence, investors prefer standard financial intermediaries when investing in real assets. Regardless, detailed empirical investigations of the first *practical* experiences can help us analyze how well these new concepts can be realized and identify their potential financial and economic consequences.

Second, we add to the literature on household portfolio choice. Goetzmann and Kumar (2008) report that conventional household equity ownership is characterized by severely under-diversified portfolios with only a few stocks, exposing households to idiosyncratic risks. Giacoletti (2021) finds that idiosyncratic risk in housing markets remains large, comprising the main investment of many households, and properties are exposed to substantial house-specific price variability. Moreover, they report that homeowners may be willing to make considerable payments to insure against idiosyncratic housing risk. Real estate property tokenization may present a solution by enabling fragmented ownership and risk sharing at the individual property level. Additionally, fractional ownership allows young investors to enter local real estate markets gradually with small monthly investments, allowing these investors to hedge the price risk until they can purchase their own residential property. Price risk remains substantial for first-time house owners as they enter the residential real estate market.

Third, we extend real estate literature. Feng et al. (2021) suggest that whether geographical diversification within real estate investment trusts is desirable or should be left to investors in these trusts remains an open question. Tokenization allows individual investors to create robust real estate portfolios instead of relying on residential real estate investment trusts (REITs), mutual funds, or exchange-traded funds wherein investors depend on fund supply and investment philosophy and bear the associated costs.^4^ Whether ownership becomes fragmented and how individuals create portfolios are essential questions, highlighting the relevance and possibly disruptive nature of tokenized real estate markets; see Wouda and Opdenakker (2019).

Our findings can be summarized as follows. First, the median number of owners per residential property token is 254. Currently, the Herfindahl–Hirschman concentration index has a 2.0% median, while only three houses have values above 10%, which suggests large dispersion in ownership for most properties. For larger properties, the number of owners and ownership concentration are somewhat higher and lower, respectively. These empirical observations demonstrate that fragmented ownership of residential properties by tokenization is not only a theoretical possibility but also a real outcome.

Second, we report that many investors hold well-diversified portfolios. Approximately 59 (22%) investors own tokens of more than 40 houses. Two hundred sixty-three 263 investors have invested more than USD 5,000 (which is 12% of the number of investors), but this figure represents 76% of the total invested value. Of the 263 investors, only 47 (18%) hold five or fewer properties, of which 28 (11%) hold only one property. Moreover, investors in tokenized real estate markets are relatively sophisticated and understand portfolio diversification well.

Third, we demonstrate that these tokens have a lively secondary market, which is partly attributable to the management company, which guarantees a buy back of a certain number of tokens against the appraisal value of the property within 10 days, and also because of investors’ option to trade with each other. In the US, property ownership and financial products legally require the “whitelisting” of participants prior to trading. During our sample period, whitelisting is possible by first purchasing a token of the specific property from the management company. This seems to be an important hurdle for further increases in liquidity, and property ownership changes once yearly on average. We find that tokens that can be traded on decentralized exchanges are about 25% more liquid. Kreppmeier et al. (2022) examine trading behavior in the same platform in more detail, extending the liquidity analysis in this paper.

Finally, we demonstrate that property prices on decentralized exchanges need a few days to process exchange rate fluctuations between the US dollar and Ethereum, the digital currency used for these tokens. Property prices seem to be approaching their economic value in longer time horizons, and this is proxied by the local house price index. These results are consistent with the explanation that the tokenization platform and its community grew during the course of our sample period, leading to increased demand that is not directly linked to these properties’ fundamental value.

The remainder of this paper is organized as follows. Section "Background and data sources" contains our background, data sources, and descriptive statistics. Section "Empirical results" contains four subsections with an empirical examination of the four research questions that we aim to answer. Finally, Sect. "Conclusion" concludes the study.

## Background and data sources

This study utilizes data from one US-based company that has focused on making US residential real estate available for investment through the Ethereum blockchain. The company is RealT and more information about its business can be found at https://realt.co. While this section provides a summary of the business model for the reader to understand the remainder of the paper, it constitutes neither an official/legal description of the business of the company nor an endorsement of their business practices.^5^

RealT purchases residential properties and tokenizes legal entities that hold the deeds of the property according to US regulations. Management, maintenance, and rent collection of the properties are outsourced to a third party. After subtracting costs, the collected rent for the specific property is paid to token holders. If a tenant defaults, the holders of the token of that property do not receive rent payments. Fractional ownership enables risk sharing among investors but also creates moral hazard and free rider problems (i.e., when investors’ financial interest is too small such that company monitoring costs become infeasible). For instance, how can small investors in these residential properties be assured that the best party is hired for the management of their property and rent collection? RealT may select the best possible third party that is best for its management, but not necessarily for the token holders of the residential property. Ross (1973), Jensen and Meckling (1976), and Leland and Pyle (1977) have examined these moral hazard issues in corporate finance literature. RealT can reduce agency costs if it has substantial ownership of the properties; hence, inefficient management will negatively affect them. However, if RealT’s stake is too large, this may negatively affect token liquidity, and small owners may also become free riders. These owners may expect that large shareholders are available to monitor RealT and that the hired management company is financially viable; see Grossman and Hart (1980), Shleifer and Vishny (1986), and Maug (1998). However, no token holders monitor the tokenization company, inefficient property management may occur to the detriment of all token holders.

Purchasing tokens is a relatively easy process (for specifics, please consult the website of RealT.^6^) First, the investor submits their personal information (e.g., passport and selfie) along with their Ethereum wallet address. Once new properties are available for sale online, investors can purchase them with the major cryptocurrencies or via credit card. The investor then receives the contract via e-mail, which needs to be signed and returned. Finally, the investor needs to determine how and in which cryptocurrency to receive the token and the rent payments. Currently, investors opt to convert their sold property or received rent back to conventional currencies; they can convert their stablecoins to their bank account through services like Binance or Ascendex.

While companies manage the tokenization process, they remain legally separate from the company holding the real estate. According to its website, this means that when the company defaults, token owners can choose another company to assume the management of the company holding the deeds of the properties. The management company typically holds about 50 of the tokens of a property to provide liquidity for decentralized exchanges; however, they are not required to co-invest in the properties it brings to the market. Token price reflects the asset value, a maintenance reserve of about 2.5%, and the fee the management company charges of 10%.

As there are legal and compliance regulations as to who can own the tokens, the wallets purchasing the tokens need to be whitelisted by the company. A property can only be whitelisted by buying at least one token of that property via the management company’s website. As each property is a separate limited liability company, each investor holds a contract with that specific company. Hence, to purchase tokens of the property in the secondary market, the wallet already must have purchased tokens of the property in the past. While this legal requirement seems important for the liquidity of the tokens, the pool of potential investors remains relatively small.^7^ A total of 13 of its tokens can be traded via *Uniswap*. For more information about the workings of Uniswap, see, e.g., Lo and Medda (2021). Alternatively, token holders can post their bids and offers on *Swapcat* and *Airswap*. Finally, management companies can buy back tokens; however, buy back is limited in quantity (up to USD 2,000) per week and is not as fast as other more immediate methods, taking up to 10 days for the buyback finalization. Transaction prices on Uniswap, Swapcat, and Airswap are determined by two parties agreeing on the transfer. Buyback is based on an appraisal value that is refreshed once yearly. Investors should realize that these token buyback guarantees can only hold as long as the company issuing them is solvent and that insolvency risk in case of a housing crisis increases with tokenized property value.

The token holder and transfer data are from *Etherscan*, which is a search and analytics platform for Ethereum, which is a decentralized smart contracts platform that contains the real estate tokens that we examine in this paper. The price of the transfer is not available unless the trade occurs via the liquidity pool of decentralized exchanges such as Uniswap, Airswap, or Swapcat. A popular website for token investors is *Coingecko*, which displays prices and volumes of six tokens that can be traded via Uniswap.^8^

Table 1 contains more information on the tokenized properties. Most property values are in the range of USD 50,000–75,000 and (initial) token prices are in the range of USD 45–60. There are some exceptions for larger properties with values around USD 500,000 and some initial token prices were USD 150. Hence, most properties are split into about 1,000 tokens, but larger ones may have considerably more. The median house value in the sample is USD 65,211. This setup means that even relatively small properties can be owned by households. The extreme risk-sharing possibility is one of the great promises of the tokenization of real estate. Table 1 also indicates that most of the properties are located in Detroit, Michigan. The first property began collecting rent in August 2019. The portfolio of properties substantially increased in December 2020, when 21 were added. The majority of properties have a rent close to USD 6, leading to an initial rental yield of 11%.

**Table 1:** Descriptive statistics of the tokenized real estate properties

Address			City	State	Code	Price	Amount	Value	Yield	Rent	Uniswap	Start date
Monte Vista	St	18,273	Detroit	MI	48221	51.45	1,300	66,885	11.15	5.74	No	1-Feb-2021
Hartwell	St	15,095	Detroit	MI	48227	51.03	1,300	66,339	11.09	5.66	No	1-Feb-2021
Fielding	St	18,466	Detroit	MI	48219	51.88	1,300	67,444	11.13	5.78	No	1-Feb-2021
Prest	St	15,770	Detroit	MI	48227	51.15	1,300	66,495	12.02	6.15	No	28-Jan-2021
S. Avers	St	1244	Chicago	IL	60623	50.00	8,000	400,000	10.25	5.12	No	28-Jan-2021
Westphalia	St	18,481	Detroit	MI	48205	48.70	1,200	58,440	11.44	5.57	No	1-Jan-2021
Ward	Ave	15,039	Detroit	MI	48227	48.66	1,200	58,392	11.27	5.49	No	28-Dec-2020
Keystone	St	19,311	Detroit	MI	48234	49.58	1,200	59,496	11.18	5.54	No	28-Dec-2020
Buckingham	Ave	4680	Detroit	MI	48224	47.82	1,200	57,384	11.15	5.33	No	28-Dec-2020
Grand	St	4061	Detroit	MI	48238	58.27	1,800	104,886	10.06	5.86	Yes	28-Dec-2020
Mitchell	St	19,163	Detroit	MI	48234	51.48	1,200	61,776	11.16	5.74	No	28-Dec-2020
Westphalia	St	19,201	Detroit	MI	48205	49.63	1,200	59,556	11.01	5.46	No	28-Dec-2020
Everts	St	9717	Detroit	MI	48224	47.78	1,200	57,336	10.96	5.24	No	26-Dec-2020
Hartwell	St	15,796	Detroit	MI	48227	50.60	1,200	60,720	11.24	5.69	No	25-Dec-2020
Bradford	St	17,813	Detroit	MI	48205	49.63	1,200	59,556	11.21	5.56	No	24-Dec-2020
Beaconsfield	St	4380	Detroit	MI	48224	51.53	1,200	61,836	11.09	5.71	No	23-Dec-2020
Saratoga	St	13,895	Detroit	MI	48205	54.35	1,200	65,220	11.19	6.08	No	22-Dec-2020
Carlisle	St	14,078	Detroit	MI	48205	50.56	1,200	60,672	11.11	5.62	No	21-Dec-2020
Rosemary	St	14,319	Detroit	MI	48213	46.67	1,300	60,671	11.31	5.28	No	19-Dec-2020
Westphalia	St	20,105	Detroit	MI	48205	46.67	1,300	60,671	11.03	5.15	No	18-Dec-2020
Ardmore	St	15,777	Detroit	MI	48227	44.34	1,300	57,642	11.04	4.89	Yes	17-Dec-2020
Gable	St	19,317	Detroit	MI	48234	44.19	1,300	57,447	11.11	4.91	No	16-Dec-2020
Kilbourne	Ave	13,116	Detroit	MI	48213	50.17	1,300	65,221	11.03	5.53	No	15-Dec-2020
Glenfield	Ave	13,114	Detroit	MI	48213	55.31	1,800	99,558	11.36	6.28	No	14-Dec-2020
Manor	St	15,778	Detroit	MI	48238	59.87	1,300	77,831	11.97	7.17	No	11-Dec-2020
Monte Vista	St	15,784	Detroit	MI	48238	49.63	1,200	59,556	11.01	5.47	No	11-Dec-2020
East 71		4340	Cleveland	OH	44105	54.37	8,000	434,960	13.08	7.11	No	1-Dec-2020
Jefferson	Ave	581	Rochester	NY	14611	58.48	10,000	584,800	10.26	6.00	No	30-Nov-2020
Somerset	Ave	10,604	Detroit	MI	48224	52.74	1,300	68,562	11.76	6.20	No	7-Nov-2020
Devonshire	Rd	9133	Detroit	MI	48224	54.49	1,300	70,837	11.84	6.45	No	6-Nov-2020
Florida	Ave	1000	Akron	OH	44314	58.99	4,000	235,960	7.45	4.40	No	12-Oct-2020
Warwick	St	13,991	Detroit	MI	48223	44.99	1,300	58,487	10.20	4.59	No	5-Oct-2020
Greenview	Ave	6923	Detroit	MI	48228	50.17	1,300	65,221	11.41	5.72	No	5-Oct-2020
Faust	Ave	18,433	Detroit	MI	48219	48.46	1,300	62,998	11.16	5.41	No	25-Sep-2020
Worden	St	10,974	Detroit	MI	48224	53.59	1,300	69,667	11.54	6.18	No	17-Sep-2020
Lansdowne	St	12,334	Detroit	MI	48224	53.59	1,300	69,667	11.80	6.32	No	14-Sep-2020
Harding	St	3432	Detroit	MI	48214	49.23	1,300	63,999	11.84	5.83	No	2-Sep-2020
Boleyn	St	9169	Detroit	MI	48224	53.64	1,300	69,732	12.09	6.48	No	20-Aug-2020
McKinney	St	10,616	Detroit	MI	48224	50.13	1,300	65,169	11.46	5.75	No	16-Aug-2020
Courville	St	9309	Detroit	MI	48224	51.07	1,400	71,500	11.92	6.09	No	4-Aug-2020
Somerset	Ave	10,612	Detroit	MI	48224	47.86	1,300	62,222	11.41	5.46	No	24-Jul-2020
Devonshire	Rd	9166	Detroit	MI	48224	53.21	1,400	74,489	12.78	6.80	No	22-Jul-2020
Grayton	St	10,084	Detroit	MI	48224	50.28	1,300	65,361	11.27	5.67	No	8-Jul-2020
Grayton	St	10,048	Detroit	MI	48224	50.73	1,300	65,944	11.91	6.04	No	3-Jul-2020
Kensington	Ave	9165	Detroit	MI	48224	52.91	1,300	68,778	12.90	6.82	No	3-Jul-2020
Freeland	St	15,048	Detroit	MI	48227	49.02	1,300	63,722	11.66	5.72	No	18-Jun-2020
NE 42nd	Crt	272	Deerfield Beach	FL	33064	57.96	3,000	173,889	7.00	4.06	No	11-Jun-2020
Liberal	St	15,634	Detroit	MI	48205	48.98	1,200	58,776	13.20	6.47	Yes	3-Jun-2020
Mansfield	St	18,900	Detroit	MI	48235	51.31	1,100	56,441	11.26	5.78	Yes	28-May-2020
Andover	Dr	25,097	Dearborn Heights	MI	48125	53.13	1,400	74,389	11.21	5.96	Yes	12-May-2020
Appoline	St	18,276	Detroit	MI	48235	52.32	1,400	73,244	12.13	6.35	Yes	12-May-2020
Schaefer	Hwy	8342	Detroit	MI	48228	50.83	4,000	203,333	12.78	6.49	Yes	16-Apr-2020
Lesure	St	20,200	Detroit	MI	48235	69.40	1,000	69,400	10.39	7.21	Yes	5-Mar-2020
Appoline	St	10,024	Detroit	MI	48227	145.56	4,000	582,200	11.47	16.70	Yes	28-Jan-2020
Patton	St	9336	Detroit	MI	48228	62.70	1,000	62,700	10.40	6.52	Yes	28-Jan-2020
Audubon	Rd	5942	Detroit	MI	48224	77.73	750	58,300	12.40	9.64	Yes	4-Nov-2019
Fullerton	Ave	16,200	Detroit	MI	48227	144.74	3,800	550,000	9.45	13.68	Yes	24-Aug-2019
Marlowe	St	9943	Detroit	MI	48227	63.75	1,000	63,750	12.90	8.22	Yes	24-Aug-2019

*Price in USD per token. Value in USD. Rent in USD per token. Yield is rent/price per token. Uniswap ‘Yes’ means that token can be traded *via* Uniswap. Date is rent starting date*

## Empirical results

In this section, we present empirical evidence on each of the four research questions that we presented in the introduction.

### How concentrated is the ownership of a typical residential property after digitization?

Digitization of residential real estate may allow investors with relatively modest wealth to diversify their portfolios. Tokenized real estate assets compete with REITs, which have been around since the 1960s in the US. In tokenization, investors do not depend on the manager of the REIT for the decision on which real estate properties are included in the portfolio. However, some investors may find it feasible to pay the management fee to outsource these decisions, while others may feel more comfortable doing their due diligence and constructing their portfolios. For these investors, tokens that represent residential real estate can be cost-effective solutions. However, it is an open question whether in practice ownership of tokenized residential properties is dispersed or concentrated. For this sample of real estate properties, we document the dispersion of ownership in two different ways. First, we indicate with a histogram how many owners the typical property in this sample has. Second, we compute the (adjusted) Herfindahl–Hirschman Index (HHI) of ownership for each property:

$$HHI={\sum }_{i=1}^{N}{s}_{i}^{2}$$

where ${s}_{i}$ is the ownership percentage of holder $i$, out of a total of $N$ owners. This index varies between 1, for maximally concentrated ownership, to $\frac{1}{N}$ when all investors have an equal share of the total value. The adjusted index eliminates the differences between properties that can arise because of a different number of owners:

$${HHI}^{*}=\frac{HHI-\frac{1}{N}}{1-\frac{1}{N}}$$

with ${HHI}^{*}=1$ when there is only a sole owner of the property. The index equals 0 when all holders hold the same fraction of the property. To better understand what the drivers are between dispersion in ownership, we regress the number of owners $N$ and ${HHI}^{*}$ on several possible factors:

$${N}_{i}\left(or {HHI}_{i}^{*}\right)=\alpha +{\beta }_{1}\cdot {NUM}_{i}+{\beta }_{2}\cdot {VAL}_{i}+{\beta }_{3}\cdot {AGE}_{i}+{\beta }_{4}\cdot {DETROIT}_{i}+{\varepsilon }_{i}$$

where *NUM* is the number of tokens the property is split in, *VAL* is the total value of the property, *AGE* is the number of days ago the token started to collect rent, and *DETROIT* is a dummy indicating whether the property is in Detroit or not. Note that this is a cross-sectional linear regression using data at the end of our sample. The advantage of a linear regression model is that the imposed structure allows for statistical inference. However, because we have a small sample and variables may be nonlinearly related to each other, there is a possibility that the model is not well specified.

The expectation is that a larger number of tokens results in more dispersed ownership. First, because the minimum unit of initial purchase is one token (afterwards it can be split), and second because psychologically it may serve as a numeraire for investors. It can also be expected that the higher the value of the property, the more owners a property may have. Typically, a more expensive property will also be divided into more tokens, but this relationship is not perfect. With respect to the age of the property, it is not entirely clear what that would mean for the number of owners. It could mean more dispersed ownership, as over time more people opt to add it to their portfolio. However, as a token can only be purchased in the secondary market after you have purchased a token via the website of the management company, this is unlikely to be an important driver. It could be that more recently, more potential investors have heard about the company and gained trust in it, leading to more interest from a wider group, leading to more dispersed ownership. Finally, as most properties are in Detroit, diversification is more limited compared to properties outside the city. This would make properties outside Detroit more attractive to a larger group of investors and the effect of the Detroit-dummy a negative effect on the dispersion of ownership.^9^

**Fig. 1:**
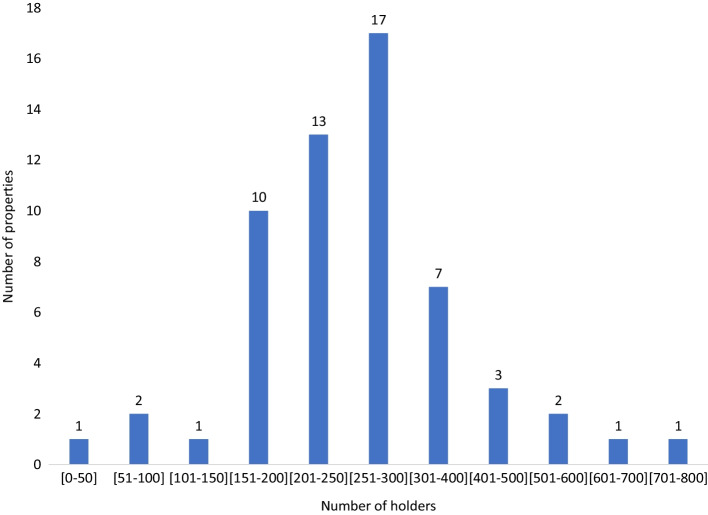
Histogram of the number of holders per property. Figure based on data from March 2021. Contains ownership of the 58 properties in Table 1

Figure 1 presents the histogram of the number of holders per property. The lowest number of holders is 15 owners, and the largest is 776. We find that the bulk of the properties have between 150 and 400 holders. As a stylistic example, for properties with a value of USD 60,000 and 200 holders, a holder would invest on average USD 300 in a property. The number of holders suggests that indeed tokenization leads to broad ownership by many small investors. However, the disadvantage of the number of holders is that we do not know how skewed the ownership is.

**Fig. 2:**
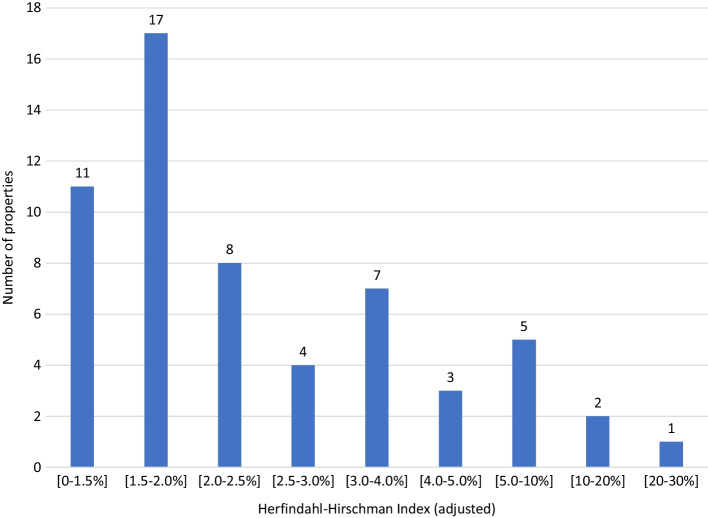
Histogram of ownership concentration per property. Figure based on data from March 2021. Contains ownership of the 58 properties in Table 1

The HHI highlights the concentration. However, it may be prudent to first get a feeling of how this number connects to ownership. For example, suppose one holder owns half the property and the other 199 together the other half in equal proportions. Hence, the adjusted HHI would be 24.8%. When 10 investors each hold 5% and 190 hold the rest in equal shares, the ratio drops to 2.1%. Figure 2 contains the ownership concentration measure. There are 28 properties with ownership concentration below 2%. Only three properties have an ownership concentration above 10%. The maximum is 26.7% and is for the property with only 15 holders, for which the largest owns half. These results further indicate that house ownership is generally not concentrated, and tokenization leads to substantial risk sharing across individuals.

Table 2 contains the estimation of the regression equations. The number of holders and concentration seem unaffected by the number of tokens issued. However, property value (which is correlated to the number of tokens) is statistically significant: the higher the value, the more owners (t-stat 2.72) and the less concentrated the ownership (t-stat-2.12) is. This is consistent with our expectations. Properties listed a long time ago have fewer holders and are less dispersed in ownership. This suggests that fewer investors were aware or confident enough to invest in these tokens when it started; however, once the tokenization business matured, more investors have been attracted. The number of owners is lower for properties located in Detroit, but ownership concentration is not different across locations.

**Table 2:** Explaining dispersion of ownership

	Number of owners (N)		Ownership concentration (HHI*)	
	Coefficient	t-statistic	Coefficient	t-statistic
Intercept	382.2	7.52*	− 0.476	− 0.15
Token amount (NUM)	0.015	1.12	0.0012	1.45
Property value (VAL)	0.00040	2.72*	− 0.000020	− 2.12*
Days since listed (AGE)	− 0.417	− 6.06*	0.022	5.08*
DETROIT dummy	− 128.3	− 3.19*	0.349	0.14
R-square (adj.)	0.792		0.286	

Statistical significance at the 5% level indicated with a *

In summary, the median number of owners per residential property token is 254, and ownership concentration is generally low. This suggests that ownership of residential properties by tokenization is fragmented in practice.

### Do token investors use fractional ownership to create diversified real estate portfolios?

Blume and Friend (1975) and Goetzmann and Kumar (2008) report that investors used to be highly under-diversified, as they only hold a few stocks in their portfolio on average. Baltussen and Post (2011) demonstrate with an experiment that investors often employ heuristic rules, such as naïve diversification of equal weight to each investable asset, instead of considering economic diversification. In this subsection, we evaluate investors’ portfolios, here represented by unique wallets. We examine the real estate token portfolio of each wallet but cannot relate it to the other assets that the owner of the wallet may have in their portfolio. We have no other characteristics of the owners of the wallets. This is a limitation of our research. However, the question of whether the real estate token portfolio itself is diversified or contains large idiosyncratic risks is still warranted.

**Fig. 3:**
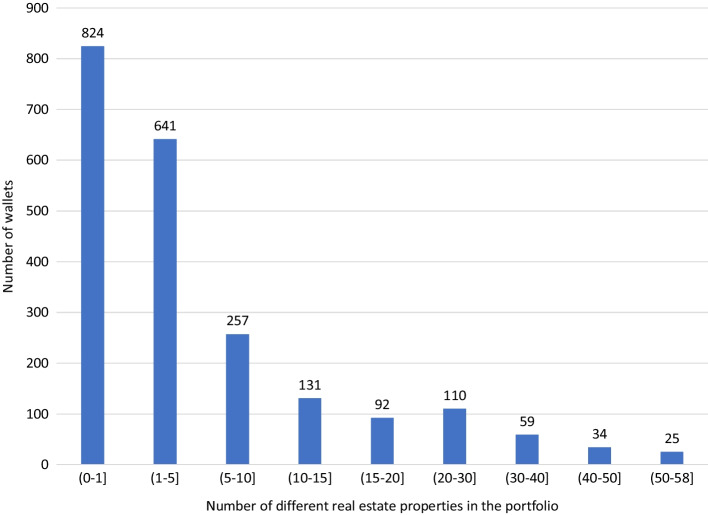
Histogram of the number of different real estate properties per portfolio. Figure based on data from March 2021. Contains ownership of the 58 properties in Table 1

Figure 3 contains the number of tokens held per unique wallet address, which we interpret as the number of unique investors, even though we cannot exclude that some investors opened two or more wallets.^10^ Note that if this is the case, we probably underestimate the diversification, as it is more likely that separate wallets would be created for ownership of different tokens rather than ownership of a certain token spread out over more wallets. We find that there are 2,173 unique wallets, of which 824 own only one token. There are 641 investors that own tokens of 2 to 5 properties. On the other end, there are 59 investors that own tokens of more than 40 (out of a maximum of 58) properties.

**Fig. 4:**
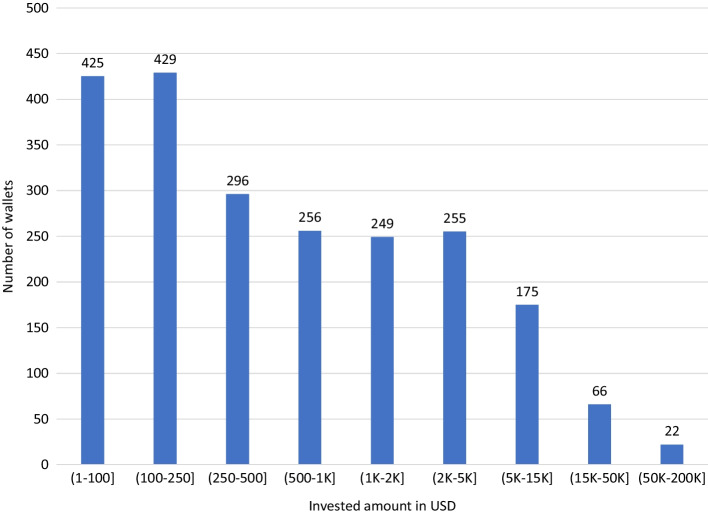
Histogram of the invested amount (in USD) per portfolio. Figure based on data from March 2021. Contains ownership of the 58 properties in Table 1

Figure 4 is a similar histogram, but it focuses on the invested amount (in USD) per investor, where we use the price of the initial token as the invested amount as we do not have other estimates of the “economic value” for all properties. However, the left sides between Figs. 3 and 4 are connected as owners of a few tokens cannot have invested a lot of money given the dispersion in property ownership that we already observed in the previous subsection. About 425 wallets have invested less than USD 100 in total, corresponding to wallets with only one of these tokens in it. There are 429 investors with between USD 100 and USD 250 invested. But several investors have acquired much larger positions, as there are 88 investors with more than USD 15,000 invested, of which 22 have invested even more than USD 50,000. The maximum investment amount is USD 174,527. Of the 263 investors that have invested more than USD 5,000, only 47 hold 5 or fewer properties (of which 28 hold only 1 property).

**Fig. 5:**
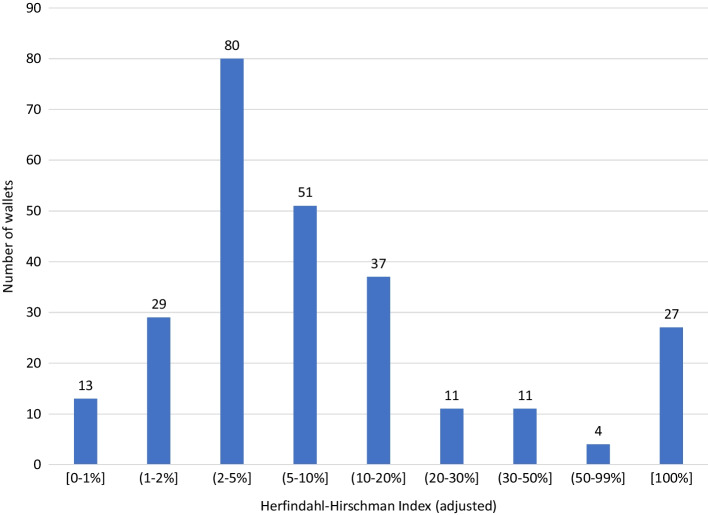
Histogram of the dispersion in holdings per portfolio (invested > USD 5,000). Figure based on data from March 2021. Contains ownership of the 58 properties in Table 1

The histogram in Fig. 5 examines the extent to which investors diversify their portfolios. Here we take the subsample of 263 investors who invested more than USD 5,000. While this is only 12% of the total *number* of investors, it accounts for 76% of the *value* of invested capital. Examining investor diversification with less invested capital is not so informative, as a small investor may have bought a few tokens of USD 50 as a trial. Moreover, the minimum initial investment is one token, so those with low invested amounts cannot have well-diversified portfolios. To examine investor diversification, we calculated the HHI* for the portfolio weights (i.e., the value of each property investment divided by the total value of the investor’s investment). If they hold an equally weighted portfolio, this measure equals 0. However, if they hold a portfolio concentrated on one property, it will be close to one. Figure 5 suggests that out of the 263 investors who invested more than USD 5,000, 42 (16%) have a portfolio that is close to equally weighted, with a concentration ratio below 2%. There are only 28 (11%) that hold only one property, and 42 (16%) investors with a concentration ratio above 30%.^11^ Even though the latter type of portfolio contains some idiosyncratic risks, it does not automatically imply an irrational portfolio choice. For example, one investor owns 14 properties with a total value of USD 132,501 and has invested USD 58,480 and USD 40,000 in two properties, with the remainder close to equally weighted in the other 12. The largest investments are in S. Avers Street in Chicago and Jefferson Avenue in Rochester, which are properties with about 10 times the average value of the others. Hence, this investor aims to own 5% to 10% of each property’s value, and not an equal dollar value of each property. Moreover, note that these two relatively large properties are not in Detroit, where most properties are located. Hence, they may represent additional *economic* diversification, as housing prices may develop differently depending on the economic situation in these cities.^12^ Hence, even investors who seem to hold under-diversified portfolios based on the concentration ratio may be more rational than one would think at first sight.^13^

Combine, we conclude that most investors with at least USD 5,000 invested hold well-diversified residential real estate portfolios.

### How liquid are individual residential properties after tokenization?

An advantage of bringing real assets to the market is that investors can buy or sell them when they have excess or shortages of liquidity. Moreover, it facilitates the efficient allocation of scarce capital through increased price discovery. An open question is to what extent the tokenization of real estate improves liquidity for its investors. As already mentioned, a disadvantage of the current regulatory setup is that the management company requires investors to be whitelisted before they can invest in a token of a property, and whitelisting can only be done by purchasing a token from the website of the management company. Basically, only investors that already own (or owned) tokens can buy more tokens than those who opt to sell them.^14^ Or the investor must sell its token back to the management company for a potentially stale appraisal-based valuation. This cumbersome whitelisting procedure that was valid during the period of our investigation has likely reduced liquidity, as potentially interested buyers on the secondary market may not have been able to acquire a token on the primary market and hence not allowed to trade.

**Fig. 6:**
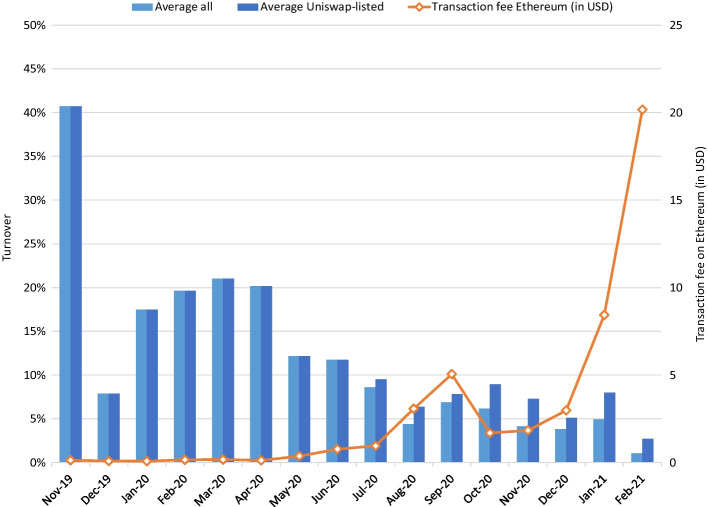
Average percentage of tokens traded. Figure based on trading data from 50 properties that were tokenized in 2019 and 2020. Average daily transaction fee of Ethereum (in USD) per month

Figure 6 indicates the average percentage of outstanding tokens traded during the month. Trades are directly derived from the blockchain and incur trading costs. Hence, it is unlikely that the liquidity reported here is caused by wash trading at certain exchanges to artificially increase their liquidity to examine competition with other exchanges, such as documented by Le Pennec et al. (2021). The average is over an expanding sample (i.e., each time a new property is tokenized it is added to the sample average). Tokens sold from the management company to the investors in primary sales are excluded from this figure. Repurchased tokens by the management are included as they provide liquidity to the token owners. As properties are tokenized on different days during the month, the first monthly observation may include only a few days and is hence omitted. Properties tokenized in 2021 are also excluded, as they would only have at most one monthly observation. Hence, the first observation in Fig. 6 is the average of trading in the tokens of two properties, while the last observation is the average of 50 properties. As tokens available to trade via Uniswap may be more liquid, we also include a column with only those tokens that can be traded on Uniswap. In the first half of the sample, average turnover is at 15% per month, indicating that a property is traded about 1.8 times per year. In the second half of the sample, liquidity is substantially lower, and with an average of about 5% turnover, a house changes hands about once every two years. Figure 6 (and in more detail in Appendix Fig. 9) indicates that the transaction fees on the Ethereum blockchain were below USD 0.5 until the middle of 2020, when they spiked above USD 3 in August 2020 and then briefly above USD 10 in September. Fees then decreased to below USD 3 toward the end of 2020. Transaction fees again increased in January and reached USD 20 per transaction in February 2021. Unsurprisingly, this coincides with the historically low turnover of residential real estate tokens.

We also aim to examine liquidity drivers. Does liquidity recede over time or does it intensify? Does liquidity improve if the token becomes available via Uniswap? To investigate this in more detail, we create monthly liquidity (turnover) figures ${L}_{i,t}$ for each property. We then treat these as observations in the following regression equation, where *UNISW* is a dummy that takes the value of 1 during the months that the token is available via that platform and *TRCST* the average daily transaction fee for Ethereum in USD in a month:

$${L}_{i,t}=\alpha +{\beta }_{1}{NUM}_{i}+{\beta }_{2}{VAL}_{i}+{\beta }_{3}{AGE}_{i,t}+{\beta }_{4}{DETROIT}_{i}+{{\beta }_{5}UNISW}_{i,t}+{{\beta }_{5}\cdot TRCST}_{t}+{\varepsilon }_{i}$$

Note that we do not use fixed or random fixed effects for a property (no ${\alpha }_{i}$ but $\alpha$) as we aim to compare liquidity across properties and not primarily how the liquidity of property varies over time. Hence, we perform a pooled regression with ordinary least squares. In an alternative specification, we include time-fixed effects (${\alpha }_{t}$ instead of $\alpha$) to exclude overall market liquidity nonparametrically. These month-fixed effects indicate how liquidity varies across properties in each month and eliminates the effect of higher liquidity until the middle of 2020, after which liquidity steadily declines. We excluded transaction fees in this regression as these do not differ across properties *i* but refer to a transaction fee on Ethereum. We prefer regressions with fixed month effects to judge the relative liquidity of properties at a given point in time but also include the regression with transaction costs and without fixed month effects to indicate that trading costs negatively affect overall market liquidity.

Table 3 displays the results of this regression. The time since the property was brought to the market presents a significantly negative effect when we excluded month-fixed effects. This may be related to the overall reduction in liquidity over time, which is not entirely captured by the nonlinear relations with Ethereum transaction fees. The impact of transaction fees on trading activity is, as could be expected, significantly negative. Moreover, the oldest properties can also be traded via Uniswap, which leads to a statistically significant (8.62 percentage points higher) liquidity, when not controlling for month-fixed effects. When including month-fixed effects, the effect of the age changes sign but is no longer significant. The number of tokens and the property value are not significant, indicating that smaller properties and those with fewer tokens are less traded. This is similar to the stock and bond market, where larger assets are more liquid. Consistent with the higher dark blue bars in Fig. 6 toward the end of the sample, we find that availability on Uniswap positively affects liquidity; however, economic size is reduced to 3.76 percentage points of additional liquidity. Whether a property is located in Detroit does not seem to affect its liquidity. Note that because of the increased Ethereum transactions costs, the company is in the process of transferring its token contracts to the Gnosis blockchain, where properties can be traded at lower cost via Levinswap, see https://info.levinswap.org/tokens.

**Table 3:** Explaining liquidity

	Liquidity		Liquidity	
	Coefficient	t-statistic	coefficient	t-statistic
Token amount (NUM)	0.41	0.65	5.10	2.99*
Property value (VAL)	− 0.004	− 0.79	4.29	2.72*
Months since listed (AGE)	− 0.59	− 3.89*	2.59	1.75
DETROIT dummy	1.02	0.57	2.44	1.71
UNISWAP dummy	8.62	7.58*	3.76	3.03*
Transaction costs	− 0.21	− 3.08*	–	–
Month fixed effects	No		Yes	
R-square	0.245		0.437	

Statistical significance at the 5% level indicated with a *

### Are prices of tokenized assets related to the economic fundamentals of the investment?

**Fig. 7:**
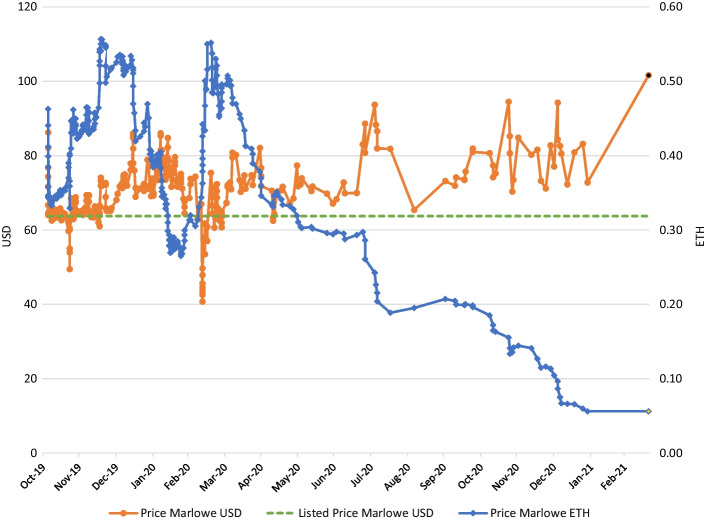
Price (in USD and ETH) of 9943 Marlowe Street Detroit MI. Each dot represents one transaction. Last colored dot is ask price on Uniswap

Another important question for financial economists is whether the price of the tokens corresponds to the “true” market price of the property, whatever that may be, or is related to other factors that are not or only remotely related to the property. In the early stages of a market, prices may not be entirely efficient and may not represent underlying asset value. For example, Dowling (2022a) finds that the prices of non-fungible tokens for virtual land in Decentraland, part of the Metaverse, are not efficient. Sebastião and Godinho (2021) report that machine learning methods may help in predicting cryptocurrency returns for three major cryptocurrencies for which a fundamental value is difficult to determine. Fortunately, we have transaction prices for the 13 real estate tokens connected to Uniswap.^15^ Figure 7 contains the price path of the first tokenized property from our sample.

The price of the tokens is quoted in ETH. Figure 7 presents that the price converted to USD stays close to the initial offering price of USD 63.75 at the beginning of the sample when many trades are available. As the exchange rate of ETH/USD is changing substantially over the sample period (Appendix Fig. 10), the price in ETH is changing considerably over the sample. Note that some large deviations from the initial offering price may be a result of large swings in the ETH/USD exchange rate that take a few days to adjust. Aharon and Demir (2022) and Umar et al. (2022) indicate that the price of non-fungible tokens does not depend on the ETH/USD exchange rate but moves exogenously, at least in the short run. This contradicts the findings of Dowling (2022b), who find some correlation between non-fungible token prices and cryptocurrency prices. Yousafet al. (2022) finds that DeFi is more correlated during the COVID-19 pandemic.

In the second half of 2020, the number of trades dropped. This decrease may be partially attributable to the increased market volume, which allows investors to buy new properties that enhance diversification instead of investing more in the same property. Moreover, note that from the fall of 2020, the token price has increased, reflecting the improved market values of the housing market in Detroit. The last dot in Fig. 7 is not a traded price, but the asking price on 20 March 2021 on Uniswap. Other properties on Uniswap exhibit very similar graphs, which can be found in the appendix.^16^ However, note that it would be unlikely for the price to drop below the initial offering price, as the management company will buy back the tokens at that price, with some limitations. From this figure and those in the appendix, we conclude that in the short run, property prices may deviate from the market value due to exchange rate fluctuations. It seems that arbitrage capital is limited and not extremely fast, but deviations do not seem to persist very long either.

**Fig. 8:**
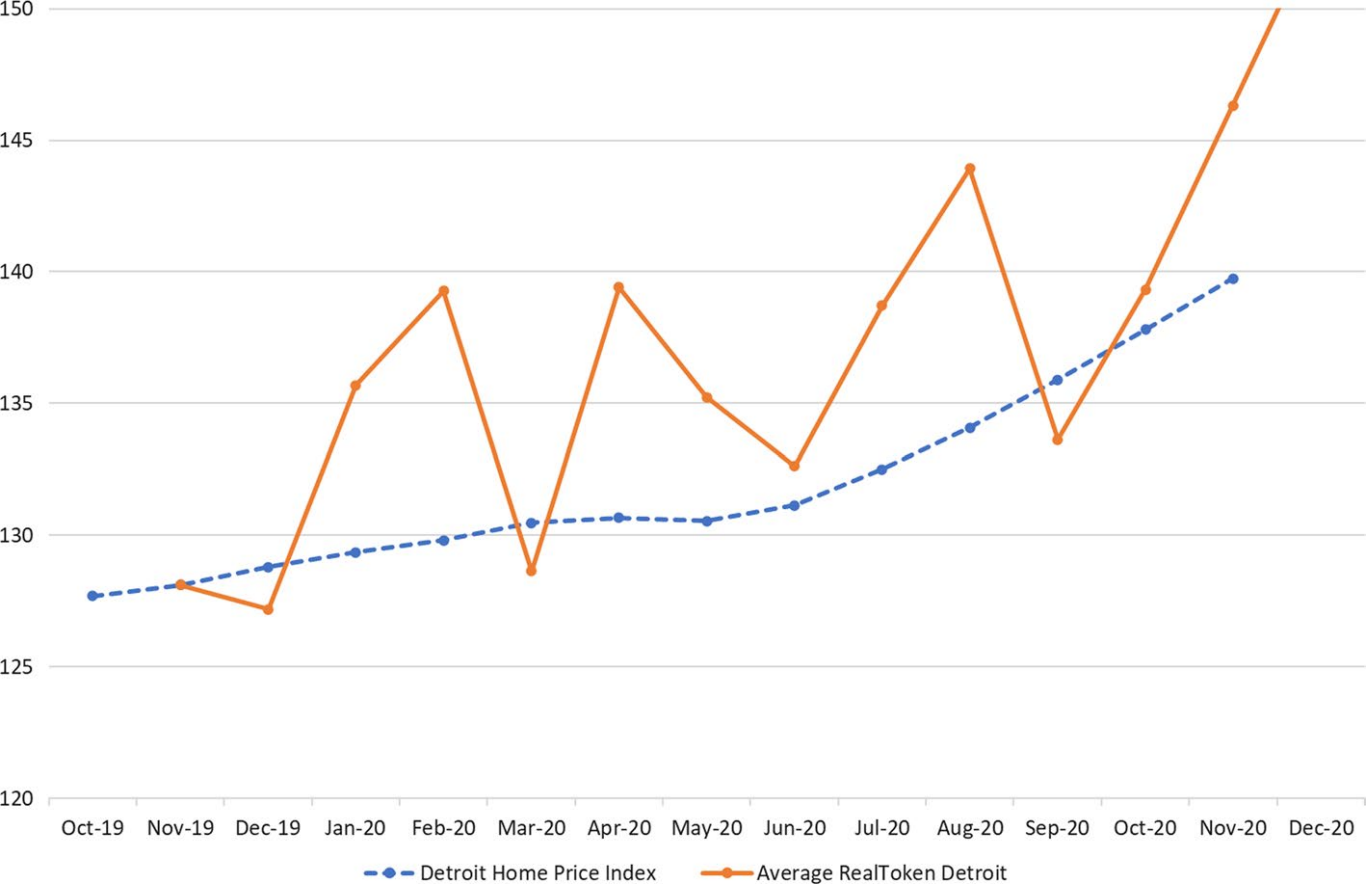
Home price index for Detroit MI and average RealToken Price. Source: S&P/Case-Shiller MI-Detroit Home Price Index, downloaded from FRED

To examine whether token prices are related to underlying economic fundamentals, we create an average RealToken Detroit Index by taking the average traded price during a calendar month, creating monthly price increases, and averaging these across properties located in Detroit. This index begins in November 2019 when the two first tokenized properties were on Uniswap. We compare the average prices of the Detroit real estate tokens with the S&P/Case-Shiller Home Price Index for Detroit MI. Figure 8 displays the comparison. The average price of the few properties in our index is more volatile and depends on the trading activity and exchange rate fluctuations of ETH/USD. Nevertheless, we find that the general pattern is similar, suggesting that token prices are indeed correlated with economic fundamentals.^17^ This finding is surprising as Fischer et al. (2021) document that even within areas with the same postal code, house price changes can differ. However, we have to remember that we are comparing average price movements here, and do not further examine the dispersion and persistence of dispersion around these averages. Additionally, other explanations may also be consistent with the increases in price data that we observe. For example, if company tokenization becomes better known, through marketing efforts or successful tokenization projects, their community may have grown. Moreover, increased demand for real estate tokens may have led to house price increases that do not directly affect the fundamental value of the underlying asset. The value of these network effects for valuation is mentioned by Nadini et al. (2021) and Cong et al. (2021). Kreppmeier et al. (2022) conclude that the crypto market sentiment may drive the valuation of real estate tokens.

## Conclusion

Our study is the first to empirically examine the financial and economic consequences of the tokenization of real estate markets using the first experiences of a 58-residential-property sample in the US. We find that tokenization fulfills its promises and leads to dispersed ownership of properties of modest value, which leads to substantial risk sharing across households. Contrary to conventional stock market portfolio selection, investors with more than USD 5,000 invested generally hold well-diversified portfolios of tokenized properties, which may reflect the higher sophistication of participants in this newly created market ecosystem. While these tokens exhibit liquidity in the secondary market, especially in those traded on decentralized exchanges, legal impediments increase it further than a turnover of once per year. We document that token trading in Ethereum adapts within a few days to fluctuations of the digital currency with the US dollar. In the longer term, token prices seem to reflect housing prices, such that portfolios of fractionally owned residential real estate properties behave like real estate investments but at a smaller scale. However, real estate token prices may not reflect the economic value of the house. Price increases remain the result of greater visibility in the tokenization platform and as a result, a larger network that is keen on investing in real estate tokens. Considering these early empirically stylized facts on tokenized real estate investments, we conclude that many of its predicted conceptual advantages can be realized. This suggests major disruptive innovations in the organization of financial markets in the future.

Our study contains the following limitations. First, our sample consists of only 58 tokenized properties, mostly located in Detroit. Despite this limited sample, a detailed analysis of the ownership distribution of these tokens and the portfolio choices made by its investors may provide us with insights into the potential and limitations of the future development of the tokenized real estate market. Second, information on the investors is limited as the real estate tokenization company (RealT) cannot share information for compliance reasons. Several other relevant research questions could be answered with access to investors’ demographic information (e.g., gender, age, nationality). Third, the researchers (and RealT) do not have information about the investors’ investment portfolios outside the wallet addresses. To gather this type of information, we would need access to, for example, the tax offices of each investor’s respective tax residency, which is generally unavailable to researchers.^18^ Unfortunately, we do not have access to these sets of information and hence cannot answer research questions involving the data.

Several directions for future research on asset tokenization seem fruitful. First, future research could examine the effect of financial regulation on the attractiveness of tokenized assets. Our data are collected from securities governed by the Securities Act of 1933 and can only be offered to US-accredited investors (Regulation D) or foreign investors (Regulation S). Hence, RealT has “know your customer” and “anti-money laundering” procedures in place. However, whether this regulatory oversight is optimal is an empirical question. Furthermore, it would be useful to compare our results with those of similar tokenization programs in different jurisdictions. Second, the evolving decentralized finance ecosystem may provide solutions to the increased transaction costs observed in periods of high gas fees on the Ethereum network. Third, whether the governance system in place can be improved is unclear. Fragmented ownership may lead to free riding, which in turn may be suboptimal for efficient property management. Finally, our proxy for house prices in Detroit is rather coarse. Estimates of tokenized residential property prices may better highlight the similarity between the token price and estimated property value.

## Figures and Tables

**Fig. 9:**
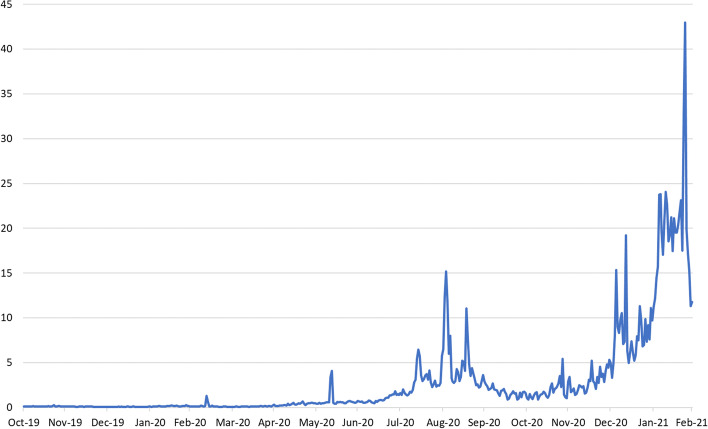
Transactions costs on Ethereum in USD. *Source*: https://blockchair.com/ethereum/charts/average-transaction-fee-usd

**Fig. 10:**
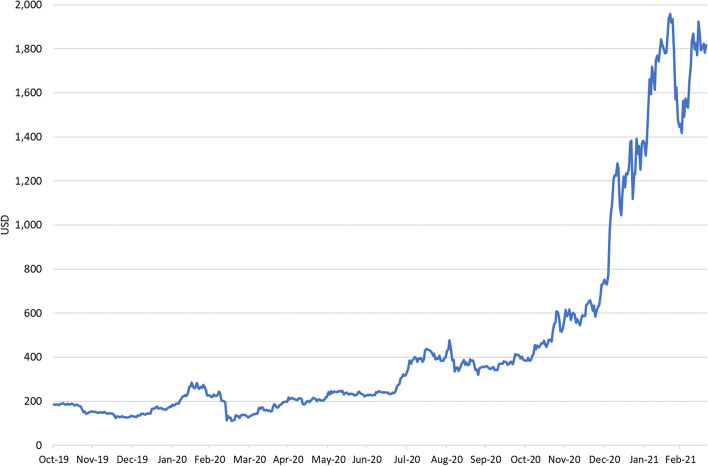
Exchange rate ETH and USD. *Source*: Yahoo Finance

**Fig. 11:**
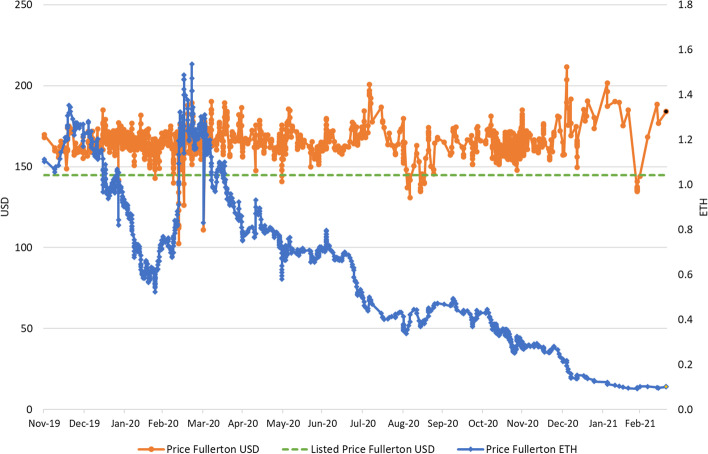
Price (in USD and ETH) of 16,200 Fullerton Avenue Detroit MI. Each dot represents one transaction. Last colored dot is ask price on Uniswap

**Fig. 12:**
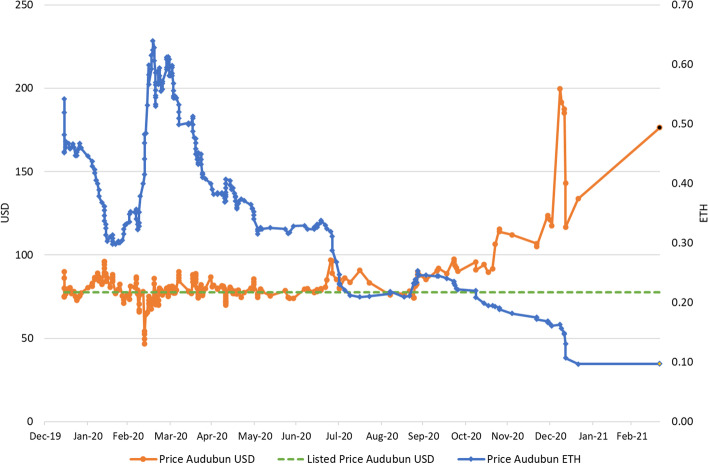
Price (in USD and ETH) of 5942 Audubon Road Detroit MI. Each dot represents one transaction. Last colored dot is ask price on Uniswap

**Fig. 13:**
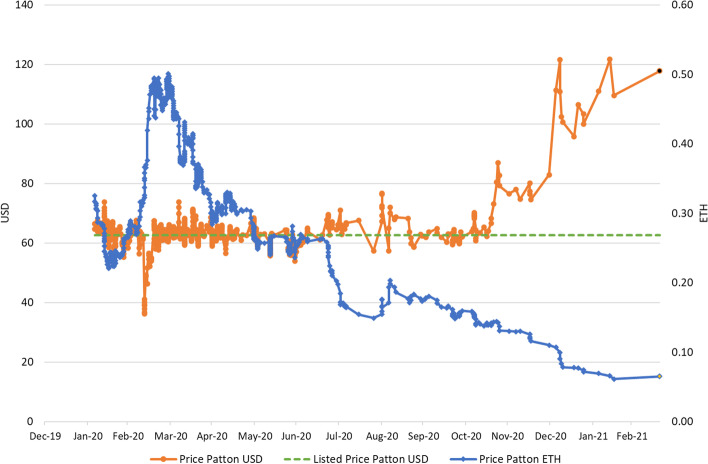
Price (in USD and ETH) of 9336 Patton Street Detroit MI. Each dot represents one transaction. Last colored dot is ask price on Uniswap

**Fig. 14:**
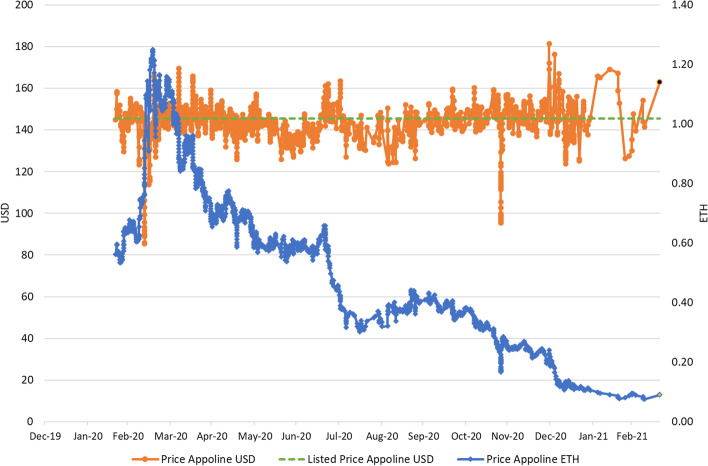
Price (in USD and ETH) of 10,024 Appoline Street Detroit MI. Each dot represents one transaction. Last colored dot is ask price on Uniswap

**Fig. 15:**
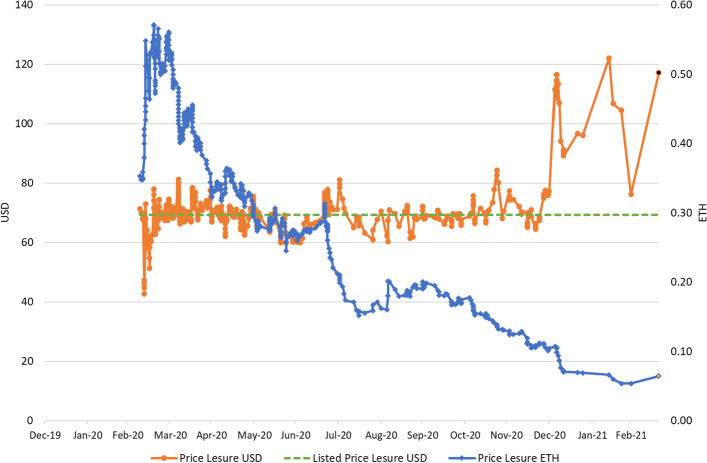
Price (in USD and ETH) of 20,200 Lesure Street Detroit MI. Each dot represents one transaction. Last colored dot is ask price on Uniswap

**Fig. 16:**
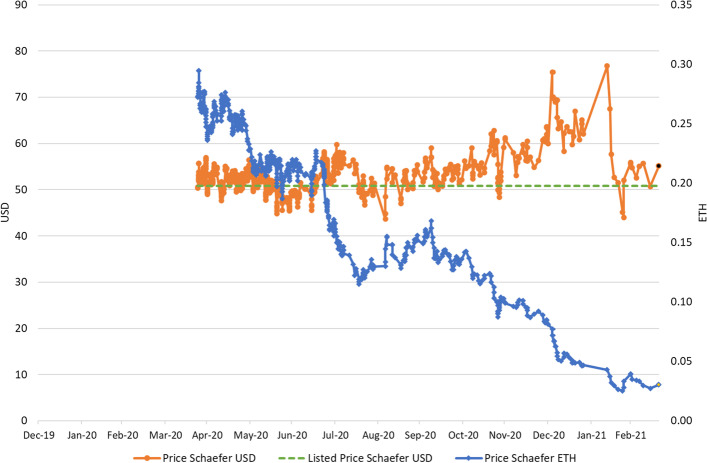
Price (in USD and ETH) of 8342 Schaefer Highway Detroit MI. Each dot represents one transaction. Last colored dot is ask price on Uniswap

**Fig. 17:**
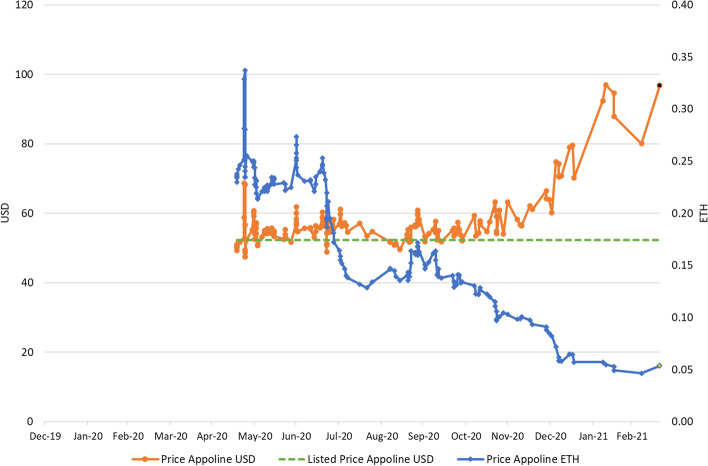
Price (in USD and ETH) of 18,276 Appoline Street Detroit MI. Each dot represents one transaction. Last colored dot is ask price on Uniswap

**Fig. 18:**
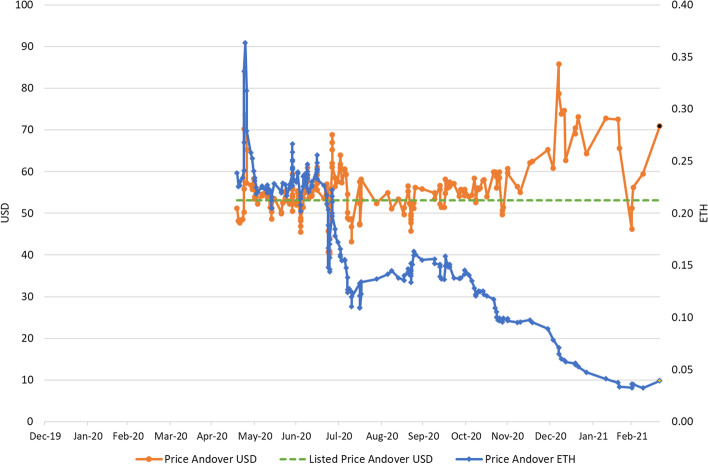
Price (in USD and ETH) of 25,097 Andover Drive Dearborn Hights MI. Each dot represents one transaction. Last colored dot is ask price on Uniswap

**Fig. 19:**
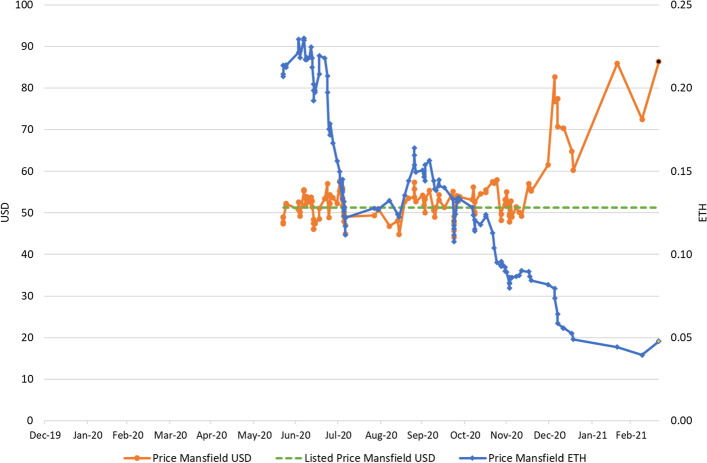
Price (in USD and ETH) of 18,900 Mansfield Street Detroit MI. Each dot represents one transaction. Last colored dot is ask price on Uniswap

**Fig. 20:**
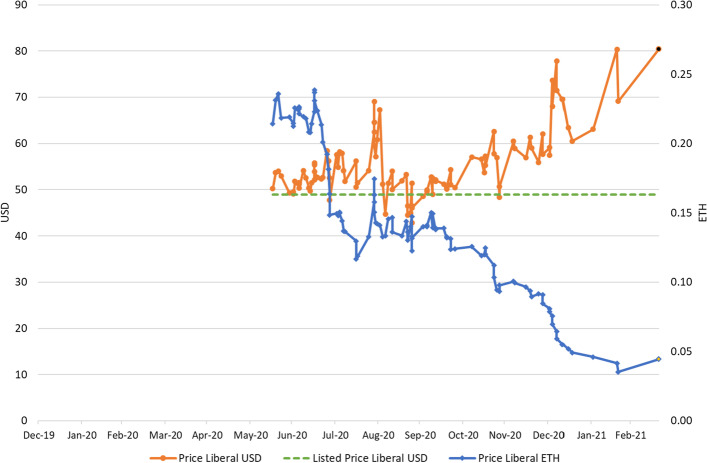
Price (in USD and ETH) of 15,634 Liberal Street Detroit MI. Each dot represents one transaction. Last colored dot is ask price on Uniswap

## Data Availability

Data is publicly available on 10.25397/eur.21666227.

## Appendix A

See Figs. 9 and 10.

## Appendix B

See Figs. 11, 12, 13, 14, 15, 16, 17, 18, 19 and 20.

## Abbreviations

HHI: Herfindahl–Hirschman Index
REIT: Real Estate Investment Trust
US: United States
USD: United States Dollar

## Footnotes

1 Several financial assets have recently become tradable on the blockchain. An example is the cryptocurrency exchange FTX, which makes it possible to trade in tokens of about 50 stocks or funds that are also available on off-chain conventional exchanges. For more information, see https://ftx.com/.

2 Forekast (2020) estimates that the value of digitized assets is about USD 18,196 million, but USD 17,725 million of this are digital currencies and not real assets. They estimate that USD 128 million of real estate has been tokenized. Another noteworthy tokenization is the USD 18 million sale of the St. Regis Aspen Resort as the security token Aspen Digital, which is part of Elevated Returns, in the fall of 2018, see https://www.aspencoin.io/ In Thailand, the SiriHub token was launched on 21 September 2021, representing ownership of one office building (Siri Campus Office Building), valued at USD 73 million; see Chow and Tan (2022). Baum (2021) lists more examples of tokenized real estate.

3 See Xu et al. (2019) for an early systematic literature review on the business and economics applications of blockchain and Fang et al. (2022) for a recent literature review on cryptocurrency trading.

4 Li et al. (2017) argue that new Fintech firms can be substitutes to traditional financial services, but also complements. It is an open question whether digital assets management would disrupt the traditional asset management industry.

5 We did not do any research into the legality or quality of the business practices of RealT, such as the probability that the smart contracts on the blockchain could be hacked or the platform or individual properties are a scam. Each investor needs to do their own due diligence. Zetzsche et al. (2019) documents that some initial coin offerings have been scams and Ponzi schemes, where investors clearly did not do their own due diligence. High-profile frauds may also affect the reputation of other, legitimate, cryptocurrencies, and blockchain-based tokens.

6 Information can be found here: https://wiki.realt.co/far/what-does-the-purchase-process-look-like-how-long-does-it-take.

7 In the meantime, the whitelisting procedure has been simplified and only registration required to purchase tokens.

8 As the trading volume of one day is often present two consecutive days on Coingecko, liquidity may seem double as large as it really is. Apart from this double counting across days, the number of tokens traded on Coingecko and Etherscan are exactly the same. The trading price per token deviates slightly, possibly due to a mismatch of the timing of the trade and the prices of the crypto currencies on the other side of the trade. We have also come across other websites that contain price and volume data on these real estate tokens, but data quality tends to be lower.

9 We do not include other property characteristics such as the floor space, number of rooms, or property type. It is likely that these affect the value of the property—which is in our regression—but there is no clear a priori reason to believe that these characteristics would influence the ownership dispersion.

10 Although no data on the identity of the owners can be shared due to legal barriers, correspondence with the tokenization firm confirmed that it is common that investors have only one Ethereum wallet for these real estate tokens. After our sample period ends, the firm also uses the Gnosis (xDai) blockchain, which means that several investors have an Ethereum and Gnosis (xDai) wallet. Since we do not examine the Gnosis (xDai) blockchain, this is not of concern for this analysis but would make any future analysis on more recent data more problematic.

11 There are 133 investors with more than USD 10,000 invested, of which 12 (9%) hold one property. The three largest investors with only one property have invested USD 73,392, USD 65,721, and USD 57,040, respectively. Note that we assume that each wallet represents a different household, but this need not be the case, so it is possible be that these investors have spread their token holdings over multiple wallets.

12 Diversification can also be examined by checking whether there are clusters of returns of token prices across different locations or types of properties; see Li et al. (2022). This form of diversification is not explored in this study because of insufficient return data (see “Are prices of tokenized assets related to the economic fundamentals of the investment?” section for an analysis of the limited available traded prices of real estate tokens).

13 Geographical diversification may also lead to information asymmetries, as the token buyer may be less familiar with the neighborhood characteristics than the seller; see Kurlat and Stroebel (2015).

14 In the meantime, the management company has simplified the whitelisting procedure, so that an investor can request whitelisting for a property by filling out a form with personal details.

15 Occasionally, the prices are also from Airswap. While there are also a few trades in the Swapcat contract, these are typically from properties not on Uniswap, which limits price discovery for individual properties to only to one or two data points. Therefore, we do not include these in this analysis.

16 The two last properties on Uniswap have recently started trading and are therefore omitted. Figures 11–20 in the Appendix B contain the graphs.

17 Our sample period is too short to provide valid conclusions about the expected return of investing in these tokens. As price seems to be related to economic fundamentals, its long-term returns may be similar to that of other residential real estate. Eichholtz et al. (2021) estimate the real return of residential real estate to be close to 5% per annum.

18 Some exceptions have been made for studies covering Finland, Norway, and Sweden where this data has been shared under strict confidentiality with academic researchers.
